# Enterohemorrhagic *Escherichia coli* O157 in milk and dairy products from Libya: Isolation and molecular identification by partial sequencing of 16S rDNA

**DOI:** 10.14202/vetworld.2016.1184-1189

**Published:** 2016-11-03

**Authors:** Aboubaker M. Garbaj, Enas M. Awad, Salah M. Azwai, Said K. Abolghait, Hesham T. Naas, Ashraf A. Moawad, Fatim T. Gammoudi, Ilaria Barbieri, Ibrahim M. Eldaghayes

**Affiliations:** 1 Department of Food Hygiene and Control, Faculty of Veterinary Medicine, University of Tripoli, P.O. Box 13662, Tripoli, Libya; 2 Department of Microbiology and Parasitology, Faculty of Veterinary Medicine, University of Tripoli, P.O. Box 13662, Tripoli, Libya; 3 Department of Food Hygiene and Control, Faculty of Veterinary Medicine, Suez Canal University, 41522 Ismailia, Egypt; 4 Department of Food Hygiene and Control, Faculty of Veterinary Medicine, Cairo University, 12211 Giza, Egypt; 5 Department of Genetics, The Lombardy and Emilia Romagna Experimental Zootechnic Institute (IZSLER), Via Bianchi 9, 25124 Brescia, Italy

**Keywords:** 16S rDNA, dairy products, enterohemorrhagic *Escherichia coli* O157, milk

## Abstract

**Aim:**

The aim of this work was to isolate and molecularly identify enterohemorrhagic *Escherichia coli* (EHEC) O157 in milk and dairy products in Libya, in addition; to clear the accuracy of cultural and biochemical identification as compared with molecular identification by partial sequencing of 16S rDNA for the existing isolates.

**Materials and Methods:**

A total of 108 samples of raw milk (cow, she-camel, and goat) and locally made dairy products (fermented cow’s milk, Maasora, Ricotta and ice cream) were collected from some regions (Janzour, Tripoli, Kremiya, Tajoura and Tobruk) in Libya. Samples were subjected to microbiological analysis for isolation of *E. coli* that was detected by conventional cultural and molecular method using polymerase chain reaction and partial sequencing of 16S rDNA.

**Results:**

Out of 108 samples, only 27 isolates were found to be EHEC O157 based on their cultural characteristics (Tellurite-Cefixime-Sorbitol MacConkey) that include 3 isolates from cow’s milk (11%), 3 isolates from she-camel’s milk (11%), two isolates from goat’s milk (7.4%) and 7 isolates from fermented raw milk samples (26%), isolates from fresh locally made soft cheeses (Maasora and Ricotta) were 9 (33%) and 3 (11%), respectively, while none of the ice cream samples revealed any growth. However, out of these 27 isolates, only 11 were confirmed to be *E. coli* by partial sequencing of 16S rDNA and *E. coli* O157 Latex agglutination test. Phylogenetic analysis revealed that majority of local *E. coli* isolates were related to *E. coli* O157:H7 FRIK944 strain.

**Conclusion:**

These results can be used for further studies on EHEC O157 as an emerging foodborne pathogen and its role in human infection in Libya.

## Introduction

Milk has played a major contribution in the human diet in many different countries across the world. Therefore, it is not surprising that considerable attention has been paid over many years to improve milk quality and in particular the hygienic quality. Bacterial contamination in milk can reduce the raw milk quality and create health hazards especially when the milk is contaminated with some certain species of bacteria with their associated enzymes and toxins that may survive pasteurization [1].

Microbiology to the dairy industry is an important issue, as recent outbreaks of food-borne illness were recorded as a result of consumption of milk and dairy products that had been contaminated with pathogenic organisms or their toxins. As a result, huge attention has been paid on the microbiological analysis of milk and dairy products to evaluate the quality and also to ensure that there are no public health hazards.

Investigations indicated that *Escherichia coli* O157:H7 is an emerging cause of foodborne illness and that young dairy cattle are a reservoir for it. A particularly dangerous type is referred to as enterohemorrhagic *E. coli* (EHEC). Infection with EHEC strains often associated with foodborne outbreaks traced to milk, dairy products, and other foods lead to hemorrhagic colitis (bloody diarrhea) and hemolytic uremic syndrome in humans [2,3].

Transmission of EHEC to humans occurs by many ways such as through consumption of undercooked meat, vegetables, and water contaminated by feces of carriers, person-to-person and contaminated environment contact. The risk of EHEC infection in humans from dairy farms can occur through the consumption of raw milk, dairy products, and contaminated meat from dairy cattle and through contamination of the dairy environment. Therefore, to reduce the risk of EHEC infection, it is important to improve the measures of control and also to enhance the management and to prevent the transmission of EHEC strains among animals, environment, and humans [4].

Libya is not an exception where there is a high demand for consumption of both milk and dairy products. It is not unusual that Libyans purchase and consume not only raw cow’s milk but also goat and sheep milk. Traditionally, camel’s milk is consumed raw neither pasteurized nor boiled. Locally made dairy products, such as soft cheeses, fermented milk, and ice cream are manufactured at small-scale dairy parlors, where hygienic measures neither applied nor enforced. Few studies on EHEC O157:H7 have been published from Libya as that linked to diarrhea in children from Libya [5] and dairy cattle in Tripoli [6].

This study was conducted to fill an existent gap in the literature about EHEC O157 isolation and molecular identification from milk and dairy products in Libya.

## Materials and Methods

### Ethical approval

No ethical approval was required as no life animals were used in this study. However, samples were collected as per standard sample collection methods.

### Collection of samples

A total of 108 samples were collected; 44 samples were raw milk (28) Cow, (9) she-camel and (7) goat, and the remaining 64 samples were locally manufactured dairy products: Soft cheeses (10 Ricotta and 21 Massora: Locally made cheese that manufactured by simple method of casein precipitation using food grade diluted organic acids), (5) ice cream and (28) fermented cow’s milk. Samples were randomly collected from different regions of Libya (Janzour, Tripoli, Kremiya, Tajoura, and Tobruk). Collected samples were transferred to the laboratory of Food Hygiene and Control Department, Faculty of Veterinary Medicine, University of Tripoli in an insulated icebox with a minimum of delay to be immediately examined for isolation and identification of existing EHEC.

### Preparation of the collected samples

The collected samples of each product were prepared according to American Public Health Association (APHA). Preparation of samples, decimal dilutions, and culturing techniques for EHEC was performed according to the methods described by the APHA [7]. Briefly, 25 mL/g from each sample was aseptically transferred into a sterile polyethylene stomacher bag and blended with 225 mL of sterile buffered peptone water (Cat. #610098, LIOFILCHEM, Italy) in a stomacher (Stomacher 400, Seaward Medicals, UK) at 230 rpm for 1 min. Serial dilutions were made using sterile 0.1% peptone water.

### Isolation and cultural characteristics of E. coli O157

A volume of 0.1 mL of appropriate dilutions was spread evenly on a dried surface of Tellurite-cefixime-sorbitol MacConkey (TC-SMAC, Oxoid, UK) agar plates [8]. All inoculated plates were incubated at 37±0.5°C for 18-24 h. Typical colonies of *E. coli* O157 were colorless or neutral/gray with a smoky center and 1-2 mm in diameter. Five typical colonies were picked and cultured on nutrient agar slants and incubated at 35±0.5°C for 18-24 h for further identification by Levine’s eosin-methylene blue (L-EMB, Oxoid, UK) Agar which shows a characteristic green metallic shine colony of EHEC O157:H7.

### Identification of E. coli O157 by polymerase chain reaction (PCR) and partial sequencing of 16S rDNA

The procedure of DNA extraction of *E. coli* O157 isolates was done in the same way as described before [9]. Partial 16S rDNA was amplified using the universal oligonucleotides primers Forward: S-D-Bact-0341-b-S-17 and Reverse: S-D-Bact-0785-a-A-21 adopted from [10]. Universal primers were used as this study was part of a project titled “Genetic authentication of bacterial isolates from meat and milk products in Libya and establishing the Food-borne Libyan-type Bacterial Collection (FLBC).” Therefore, the entire project was not focused on *E. coli* but also for many other bacterial isolates (total of 330 PCR products were sequenced) from milk and meat products in Libya; hence, the universal primers were used to detect all other bacterial isolates in all samples. The amplified 16S rDNA PCR fragment (464 bp) was excised from the gel, and the DNA was extracted from the gel using GF-1 Ambi Clean kit (Cat. #GF-GC-100, Vivantis, Malaysia). The purified 16S rDNA amplicons were then sequenced in Istituto Zooprofilattico Sperimentale della Lombardia e dell Emilia Romagna, Brescia, Italy, as described before [9]. All sequences will be submitted to the GenBank in the near future once completing of all project studies.

BLAST search was carried out for the obtained consensus sequences by both NCBI (http://www.ncbi.nlm.nih.gov/pubmed) and 16S bacterial cultures Blast Server for the identification of prokaryotes (http://bioinfo.unice.fr/blast/).

Latex agglutination test (*E. coli* O157 latex test; Oxoid Ltd.) was used for confirmation of existence of *E. coli* O157.

Phylogenetic tree was constructed using neighbor-joining algorithm (http://www.phylogeny.fr/simple_phylogeny.cgi) of partial 16S rDNA nucleotides sequence to show the phylogenetic relationships between local isolates sequences and those between the sequences of *E. coli* O157: H7 (Sakai and FRIK944 strains), *Shigella flexneri*, *Salmonella* Typhi, *Proteus vulgaris*, and *Pseudomonas aeruginosa* available in the GenBank, the National Institutes of Health genetic sequence database.

## Results

Out of the 108 cultured samples, only 27 (25%) yielded bacterial growth on TC-SMAC suggesting EHEC O157 (Table-1). With the exception of ice cream samples that revealed no growth on TC-SMAC, other samples showed differences in the recovery rate of suspected colonies; the lowest recorded in goat’s milk where only 7.4% (2/27) showed colonies suspected to be EHEC O157, while the highest rates of isolation recorded in Massora cheese 33% (9/27) followed by fermented cow’s milk 26% (7/27) (Table-1). Only 11% (3/27) in each of the raw cow’s milk, raw she camel’s milk and Ricotta samples had growth on TC-SMAC with colonies suspected to be EHEC O157.

**Table 1:** Isolation and molecular identification of suspected EHEC in processed samples.

Type of sample	Number of samples	Number of suspected EHEC growth on TC-SMAC (%)	Number of positive EHEC by 16S rDNA sequencing (%)
Raw cow’s milk	28	3 (10.7)	1 (3.5)
Raw she camel’s milk	9	3 (33.3)	None
Raw goat’s milk	7	2 (28.6)	2 (28.6)
Fermented cow’s milk	28	7 (25)	6 (21.4)
Maasora cheese	21	9 (42.9)	2 (9.5)
Ricotta cheese	10	3 (30)	None
Ice cream	5	0 (0)	-
Total	108	27 (25)	11 (10)

EHEC=Enterohemorrhagic *Escherichia coli*, TC-SMAC=Tellurite-cefixime-sorbitol MacConkey

**Figure-1:**
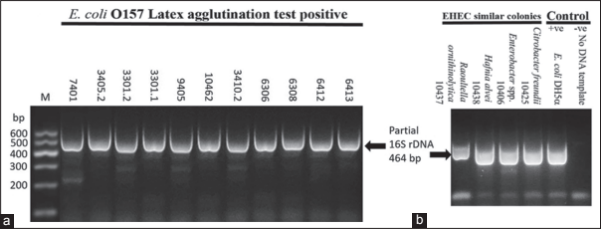
Polymerase chain reaction (PCR) amplification of 16S rDNA (464 bp) of typical bacterial colonies primarily cultivated on tellurite-cefixime-sorbitol MacConkey (TC-SMAC) and purified on Levine’s eosin-methylene blue (L-EMB) agar media. (a) Typical colonies identified as *Escherichia coli* and agglutinated with anti-O157 latex test. (b) Non *E. coli* colonies that showed similar morphological characteristic on TC-SMAC and purified L-EMB agar media. Positive and negative controls were included. The PCR products were subjected for electrophoresis in 2% agarose gel incorporated with gel red stain; the identification was based on sequencing of the purified PCR bands and BLAST search.

Bacterial isolates recovered (27) from growth on TC-SMAC were subjected to molecular analysis by DNA extraction followed by partial sequencing of their 16S rDNA. Results of sequence analysis (Table-2) showed that only 11 isolates (40%) were EHEC (Table-2 and Figure-1). Among those 11 EHEC isolates: 6 (46%) were recovered from fermented cow’s milk, 3 (23%) from raw cow’s milk, while both raw goat’s milk and Maasora gave 2 (15.4%) isolates each (Tables-1 and 2). While none was recovered from the raw camel’s milk, Ricotta and ice cream samples. That would result in an overall isolation rate of 10% (11/108) EHEC O157.

**Table 2:** Identification of suspected EHEC by PCR and partial sequencing of 16S rDNA.

Nucleotide identity % with *E. coli* O157	Isolates code	Type of sample	Sampling area
99	6413	Fermented cow’s milk	Janzour
99	6412	Fermented cow’s milk	Janzour
99	6308	Raw cow’s milk	Janzour
100	6306	Raw cow’s milk	Janzour
100	3410.2	Fermented cow’s milk	Kremiya
100	10426	Maasora cheese	Tajoura
99	9405	Fermented cow’s milk	Tobruk
99	3301.1	Raw goat’s milk	Tripoli
99	3301.2	Raw goat’s milk	Tripoli
99	3405.2	Maasora cheese	Kremiya
99	7401	Fermented cow’s milk	Tripoli

EHEC=Enterohemorrhagic *Escherichia coli*, PCR=Polymerase chain reaction, *E. coli=Escherichia* coli

**Figure-2:**
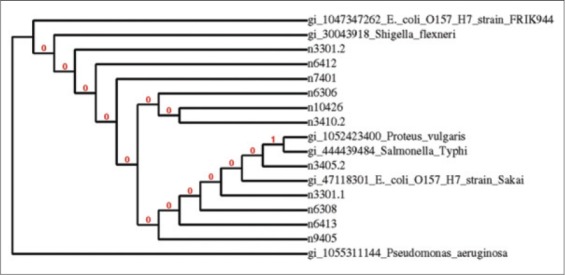
Phylogenetic tree was constructed using neighbor-joining algorithm of partial 16S rDNA nucleotides sequence showing phylogenetic relationships between the sequences of the local *Escherichia coli* isolates sequences and *E. coli* O157:H7 (Sakai and FRIK944 strains) available in databanks.

*Citrobacter freundii*, *Hafnia alvei*, *Raoultella ornithinolytica*, and *Enterobacter* spp. revealed similar cultural morphology of EHEC on TC-SMAC and L-EMB media, and the correct identification was unraveled by application of 16S rDNA PCR and partial sequencing (Figure-1). The results of the phylogenetic analysis showed that the strains 3301.2, 6412, 6413, 7401, 9405, 6306, 10426 and 3410.2 are closely related among them and with respect to *E. coli* O157:H7 FRIK944 strain with more than 99% similarity values. Meanwhile, strains 3301.1 and 6308 are related to *E. coli* O157:H7 Sakai strain (Figure-2 and Table-2).

## Discussion

A markedly large number of people are consuming raw unpasteurized milk and products made from it. Enhanced nutritional qualities, taste, and benefits have all been advocated as reasons for increased interest in raw milk consumption. However, science-based data to substantiate these claims are limited [11]. Food-borne outbreaks due to consumption of dairy products constitute a chronic problem facing food hygienists, as milk and dairy products are subjected to different sources of contamination by many pathogens either from endogenous origin or directly and indirectly from exogenous origin. The origin of contamination by food-borne pathogens varies with the type of product and the mode of production and processing. Treatment and processing of milk inhibit or encourage the multiplication of such organisms as all the nutritional components that make milk and dairy products an important part of the human diet, also support the growth of these pathogenic organisms [1,12,13].

Results obtained in this study showed that EHEC O157 has been isolated and identified from most of the examined milk and dairy products samples except from raw camel’s milk, Ricotta and ice cream samples, as refereeing to positive results confirmed sequencing of by 16S rDNA (Table-1). In our results, the percentage of suspected EHEC growth on TC-SMAC was 25% (27/108), whereas confirmation by sequencing 16S rDNA yielded 10% (11/108). Discrepancies between the isolation rates of bacteria by conventional methods and those by molecular identification (PCR and 16S rDNA sequencing) have been reported earlier [9,14].

In this study, the isolation of EHEC from raw milk samples could be attributed to fecal contamination during the milking step [15]. On the other hand, the absence of EHEC in ice cream samples in this study is most likely due to the use of reconstituted milk powder or ready to use ice cream formulas rather than fresh milk.

All the 27 positive milk and products samples tested showed growth of colonies with a metallic green sheen on Levine EMB agar, which was highly suspicious for *E. coli*. These colonies were cultured on TC-SMAC, the recommended method for the isolation of *E. coli* O157.

EHEC O157 did not ferment sorbitol and form colorless colonies. Detection of *E. coli* O157 on this medium had a sensitivity of 100% and specificity of 85% and recommended it as a simple, inexpensive and reliable means of screening *E. coli* O157 [16].

In this study, EHEC O157 was confirmed in 21.4% (6/28) of fermented cow’s milk; this is a high rate of isolation if compared to 9.6% (5/52) which was reported in Nigeria [17]. The existence of EHEC in fermented milk samples could be explained by the acid tolerant property of the organism [18]. Our data revealed that none of the raw she camel’s milk samples were positive for EHEC, however, despite scarcity of information about isolation of EHEC from she camel’s milk, one study from Iran demonstrated that 6.8% (3/44) of she camel’s milk samples collected were positive for EHEC [19]. Our results also showed that 28.6% (2/7) of EHEC isolates were recovered from raw goat’s milk samples; this is to the contrary of another study where the incidence of EHEC O157 in goat’s milk was 0.7% (3/460) in samples collected in Greece [20], while no *E. coli* O157 was detected in any of the cheeses made with raw ewe’s and goat’s milk samples from Portugal [21]. Raw cow’s milk yielded 3.5% (1/28) of EHEC O157 positive samples examined in this study. However, the percentage of positive raw cow’s milk samples with EHEC O157 in other studies were 17.4% [22], 12% [23], 10% [24], 2% [17], and 1.4% [25].

Besides the risks associated with consuming raw milk, there are concerns over the safety of cheeses made from raw milk. Among soft cheeses only Maasora yielded 9.5% (2/21) of EHEC O157 isolates recovered in this study, this result could be linked to the process of preparing Maasora, since the milk used in this type of cheese is raw without heat treatment. However, the presence of EHEC O157 in locally made fresh soft cheese samples was 6% in a study conducted in Nigeria [17]. There have been food poisoning outbreaks linked to raw milk cheeses, some of these outbreaks were due to pathogenic *E*. *coli* [26]. On the other hand, none of the Ricotta samples were positive in this study.

It has been established that consumption of raw milk or its products directly is a high risk and that *E. coli* O157:H7 can cause severe disease and even death [27,28]. In the U.S., the Centers for Disease Control and Prevention estimates that EHEC O157:H7 causes approximately 73,000 illnesses, 2000 hospitalizations, and 50-60 deaths each year [29]. In Libya, it is common and widely practiced to manufacture dairy products from raw milk. Isolation of *E. coli* O157 from milk and dairy products examined in this study could be associated with human diseases following their consumption. However, it is not possible to link our findings with any of the food poisoning cases that were occurred in Libya because of the lack of proper documentation of such cases.

## Conclusion

Findings of this study highlighted the need to improve and implement the hygienic practices related to dairy production and to apply the Libyan standards of such products for effective monitoring throughout from production to delivery. Due to the importance of the research, more work on EHEC antibiotic resistance is still ongoing. Moreover, further research is needed to fully study the incidence, prevalence and impact of toxins produced by *E. coli* and other harmful microorganisms.

## Authors’ Contributions

AMG, SMA, SKA, AAM, FTG, and IME designed and planned this research work. AMG, EMA, SMA, SKA, HTN, AAM, FTG, and IME were involved in the research by collecting samples and doing the lab work. IB has carried out the sequences of the PCR products in her lab. All authors contributed equally in preparation and revision of the manuscript. All authors read and approved the final manuscript.
